# Role of epigallocatechin-3- gallate in the regulation of known and novel microRNAs in breast carcinoma cells

**DOI:** 10.3389/fgene.2022.995046

**Published:** 2022-10-06

**Authors:** Shrila Banerjee, Abul Kalam Azad Mandal

**Affiliations:** Department of Biotechnology, School of Bio Sciences and Technology, Vellore Institute of Technology, Vellore, Tamil Nadu, India

**Keywords:** breast cancer, microRNA, EGCG, NGS study, signaling pathway

## Abstract

Breast cancer comprises 30% of all cancer cases among the world’s women population. MicroRNAs are small, endogenous, non-coding RNAs that regulate cell proliferating and apoptotic pathways by modulating expressions of related genes. Phytochemicals like epigallocatechin-3-gallate (EGCG) are known to have a chemotherapeutic effect on cancer often through the regulation of microRNAs. The aim is to find out the key known and novel miRNAs, which are controlled by EGCG in breast cancer cell line MDA-MB-231. Next-generation sequencing (NGS) revealed 1,258 known and 330 novel miRNAs from untreated and 83 μM EGCG (IC50 value of EGCG) treated cells. EGCG modulated 873 known and 47 novel miRNAs in the control vs. treated sample. The hypothesis of EGCG being a great modulator of miRNAs that significantly control important cancer-causing pathways has been established by analyzing with Kyoto Encyclopedia of Genes and Genomes (KEGG) and Protein Analysis Through Evolutionary Relationships (PANTHER) database. Validation of known and novel miRNA expression differences in untreated vs. treated cells was done using qPCR. From this study, a few notable miRNAs were distinguished that can be used as diagnostics as well as prognostic markers for breast cancer.

## Introduction

According to recent global cancer statistics, female breast carcinoma became first among the most occurring carcinoma with 2,261,419 newly diagnosed cases and 684,996 new death cases reported in the year 2020 (Sung et al., 2021). For a long time, the treatment regime against breast cancer as well as any other cancer was solely dependent upon chemotherapy, radiation therapy, tumor removal surgery, hormonal therapy, targeted drugs, and combination therapy. Due to the advancement of breast cancer treatments, the death rate declined by about 40% from 1989 to 2017 but the pace of decreased graph got reduced over the years despite the numerous dynamic therapies (DeSantis et al., 2019), thus, the need for novel therapeutic approaches increased. The major categorization of breast cancer is based upon the expression pattern of immuno-molecular markers such as estrogen receptor or ER, progesterone receptor or PR, and human epidermal growth factor receptor 2 (HER2). The percentage of breast cancer with an expressed hormonal receptor is high and because of this reason, the clinical developments in breast cancer treatment are mostly hormonal receptor-directed therapy (Roodi et al., 1995). Triple negative breast cancer or TNBC (ER-, PR- and HER2-) is the most invasive one and it also exhibits a low rate of prognosis and higher proliferation (Dent et al., 2007; Rakha et al., 2007). For the treatment of tricky and aggressive breast cancer, a novel and inventive treatment perspective is the need of the day.

MicroRNAs (miRNAs) are 18–25 nucleotide long, non-coding, endogenous, single-stranded RNA which assist in gene regulation that participates in various cell signaling pathways involved in cell growth, proliferation, differentiation, survival, and apoptosis (Xu et al., 2004; Wang et al., 2007). As carcinogenesis is similar to eukaryotic cell division, growth, and development, miRNAs are also capable of regulating oncogenesis like normal cell growth and they are characterized into oncogenic and tumor-suppressor miRNAs upon their mode of regulation (Zhang et al., 2007). miR-21 is known for its oncogenic nature in various cancers like breast (Wang et al., 2019), lung (Yanaihara et al., 2006), stomach (Zhang et al., 2008), prostate (Folini et al., 2010), colon (Slaby et al., 2007) ovarian (Tang et al., 2020), liver (Bharali et al., 2019) cancer, etc. Apart from miR-21, the miR-17-92 cluster is also a known oncogenic miRNA which often up-expressed in breast (Moi et al., 2019), lung (Zhang X. et al., 2018), lymphomas (Fassina et al., 2012), colon (Tsuchida et al., 2011), liver (Gong et al., 2018) cancer, etc. On the contrary, let-7 and miR-34 family are known tumor suppressor miRNAs that regulate breast (Liu et al., 2015; Imani et al., 2018), lung (Takamizawa et al., 2004; Sun et al., 2021), colon (Roy et al., 2012; Chang et al., 2019), liver (Jin et al., 2016; Zhang H.-F. et al., 2018), prostate (Nadiminty et al., 2012; Hagman et al., 2013), ovarian (Li et al., 2015; Biamonte et al., 2019) cancer, etc. Apart from these, altered expressions of different miRNAs are found in different cancers. For example, miR-125b is one of the prominent biomarkers of ovarian as well as thyroid cancers which is known to control cell proliferation and induce apoptosis (Nam et al., 2008; Visone et al., 2016) but in prostate cancer, the same miRNA act oncogenic by negatively regulating p53 gene (Le et al., 2009). Circulating miRNAs have become popular in the detection of various cancers like lung, breast, thyroid, colorectal, gastric cancer, etc. Differential expressions of miR-25 and miR-223 were first investigated in serum samples of non-small cell lung carcinoma patients (Chen et al., 2008). In the case of pancreatic cancer, miR-225 has often been considered a prominent serum biomarker where it shows higher expression. (Kawaguchi et al., 2013). As it was mentioned before, miRNAs like miR-21, let-7, and miR-34 are often differentially regulated in breast cancer, other notable miRNAs are also related to it. miR27a often promotes cancer growth in triple negative breast cancer by regulating the Wnt pathway (Wu et al., 2020). miR-19b suppresses E-cadherin and promotes ICAM-1 and Integrin β1 causing breast cancer progression (Seguin et al., 2015). Breast cancer with irregular miRNA expression can act as a therapeutic target. Having a narrow therapeutic index, most chemotherapeutic drugs have higher side effects; natural phytochemicals were scientifically proven to have high anti-cancerous activity with lesser side effects (Zhang et al., 2015).

Green tea, the dried and unfermented leaves of the tea plant (*Camellia sinesis*), is one of the popularly known and frequently consumed non-alcoholic beverages in the Asian continent. Green tea is rich in anti-inflammatory, anti-oxidant, and anti-cancerous polyphenolic phytochemicals particularly the epigallocatechin-3-gallate (EGCG) (Khan and Mukhtar, 2007). *In-vitro* and *in-vivo* studies showed that EGCG is capable of stimulating cell cycle arrest as well as apoptosis by inhibiting cell proliferating pathways and promoting apoptotic pathways (Khan et al., 2006). The effect of EGCG in the regulation of breast cancer was established when EGCG was found to regulate hormonal pathways (Goodin et al., 2002). EGCG modulates Bax and p53 to induce apoptotic pathways in breast cancer through combinational approaches with commercial chemotherapeutic drugs (Roy et al., 2005). It is also known that EGCG is capable of regulating different diseases including cancer through modulation of miRNA expression profile. Research showed that miRNA expression in cancer is often regulated by EGCG. The miRNA Let-7 gets up-expressed through activation of the laminin receptor by EGCG (Yamada et al., 2016). EGCG upregulates miR-210 which controls and stabilizes transcription factor HIF-1α causing suppression in lung cancer progression (Wang et al., 2011). The apoptotic effect has been observed in hepatocellular carcinoma due to the downregulation of Bcl-2 by EGCG modulated miR-16 (Tsang and Kwok, 2010). EGCG often regulates miRNAs in breast cancer, e.g., it downregulates miR-15 which decreases cancerous growth and invasion (Zan et al., 2019). A major percentage of breast cancer with expressed hormonal receptors also get suppressed due to EGCG modulated miRNAs (Fix et al., 2010). Differential miRNA expression profiles between cancer samples and EGCG treated sample scans give more insight into the relationship between miRNA and carcinogenesis. NGS study using A549 lung cancer cells and EGCG-treated A549 lung cancer cells showed that EGCG can modulate various cancer-related pathways by altering different miRNA expressions (Bhardwaj and Mandal, 2019).

The objective of the present study is to investigate the effect of EGCG on miRNA expression profile in the breast cancer, recognize targeted genes and understand cancer-related pathways modulated by differentially expressed miRNAs. Furthermore, expression validation of selective known and novel miRNAs is also studied.

## Materials and methods

### Material

EGCG and human miRNA primers were purchased from Sigma Chemical Co. St. Louis, Missouri, United States, USA. Dulbecco’s modified eagle medium with high glucose [DMEM], fetal bovine serum [FBS], MTT, and AO/EB stain were purchased from HiMedia, India. Primers for has-miR-21-3p, has-miR-320a, has-let-7e-3p, has-miR-27a-3p, and novel miRNA were purchased from Qiagen, Hilden, Germany. The human breast cancer cell line MDA-MB-231 was procured from National Centre for Cell Science, Pune, India.

### Cell culture

The MDA-MB-231 cells were cultured in Dulbecco’s modified eagle medium with high glucose [DMEM] containing 1X antibiotic-antimycotic solution and 10% fetal bovine serum (FBS) at 37°C with 95% humidified atmosphere and 5% CO2. After 80%–90% confluency, MDA-MB-231 cells were treated with the desired concentrations of EGCG for 24 h.

### Cell viability assay

MDA-MB-231 cells were seeded (10,000 cells per well) in 96-well plates and allowed to grow for 24 h. MDA-MB-231 cells were treated with different concentrations of EGCG (from 0 to 100 µM concentration) and incubated for 24 h. Afterward, 10 μl of MTT [3-(4, 5-dimethylthiazol- 2-yl)-2, 5- diphenyltetrazolium bromide] and 200 μl of 5 mg/ml PBS (phosphate buffered saline) solution were added to each well and incubated at 37°C for 4 h. After dissolving the formazan crystals in the DMSO and added to each well, a microplate reader was used to read the absorbance of each well at 570 nm. The linear regression analysis was applied to estimate the half-maximal inhibitory concentrations (IC50) from the *in vitro* dose-response curves.

### Next-Generation Sequencing (NGS)

MDA-MB-231 cells treated with 83 μM of EGCG for 24 h, and untreated MDA-MB-231cells (as control) were used for NGS. After 24 h of culture, RNA was extracted from the cells using TRizol reagent (Takara Bio Inc., Kusatsu, Japan) following the manufacturer’s protocol. The small RNA library construction and deep sequencing were carried out at Genotypic Labs Pvt. Ltd., Bengaluru, Karnataka, India. For library construction, Illumina Nextseq Single-end sequencing was used. The raw read counts and the normalized files were submitted to NCBI (National Center for Biotechnology Information) by accession number PRJNA527701.

### Classification and differential expression analysis of microRNAs

miRNA reads were mapped using Bowtie referring to the human genome (GRCh38). Identification of known miRNAs was done using the miRBase-21 database by the miRNA sequence similarity approach. The sequences were checked for other ncRNA (rRNA, tRNA, snRNA, snoRNA, and piRNA) contamination. MiReap_0.22b was used to evaluate the novel miRNA prediction (Ji et al., 2013) and the mFold online application was used to predict secondary hairpin structures (Zuker, 2003). Expressed reads for each miRNA were calculated and the DESseq R software package was used for differential expression analysis. Differentially expressed miRNAs in control vs. 83 μM EGCG treatment were determined by their expression in each sample (Anders and Huber, 2010). The expressed reads in untreated control and 83 μM EGCG treatment was used to calculate the log2 fold change of expression between untreated control and 83 μM EGCG treated cells. Novel miRNAs have been given IDs to identify them.

### Validation of microRNAs (miRNAs)

Expression validation of some significant known and putative novel miRNAs was done after the extraction of total miRNA using miRNeasy Mini Kit (Qiagen Sciences, Germantown, MD, United States) from untreated control and 24 h EGCG-treated (50, 83, and 150 μM) MDA-MB-231 cells. First-strand cDNA was synthesized using the miScript PCR starter kit (Qiagen Sciences, Germantown, MD, United States) and SYBR green was used for qRT-PCR (Bio-Rad, United States). The log2 fold change was calculated using the ΔΔCT method (Livak and Schmittgen, 2001). The comparative analysis of qRT-PCR and NGS was done to validate the miRNA expression profile obtained by NGS. The log2 fold change obtained by qRT-PCR of the known miRNAs namely hsa-miR-21-3p, hsa-miR-27a-3p, hsa-let-7e-5p, and hsa-miR-320a was calculated and compared with the log2 fold change obtained in the NGS sequencing data. The log2 fold change of the putative novel miRNA EGCG-MDAMB231-4 obtained by qRT-PCR and NGS were also compared for sequence validation.

### KEGG and PANTHER pathway enrichment of targets of validated microRNAs

TargetScan and miRDB were used to execute the target prediction of known miRNAs (Rane et al., 2015), and The Database for Annotation, Visualization, and Integrated Discovery (DAVID V6.7) was used for the analysis of pathways. Moreover, miRanda software (Riffo-Campos et al., 2016) was used to carry out the target prediction of the putative novel miRNA sequences.

### Statistical analysis

Statistical analysis was performed with a *t*-Test. The experimental data were represented as mean ± SD. The results were considered significant when *p* < 0.005 or *p* < 0.05.

## Result

### EGCG induced cell cytotoxicity

**FIGURE 1:**
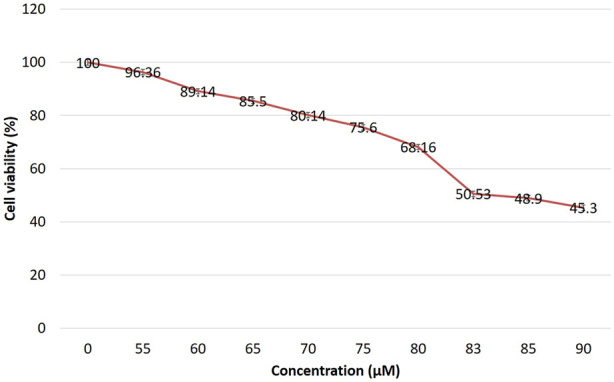
Effect of EGCG on the viability of MDA-MB-231 cells. Almost 50% of cells are viable at 83 µM EGCG treatment after 24 h. Data are presented as mean ± SD.

The inhibition of breast cancer growth rate is depicted in Figure 1. Cytotoxic effect of EGCG against MDA-MB-231 cells was checked by MTT assay. The IC50 dose level of EGCG was determined after 24 h of treatment from fitted response curves. Each curve describes how the percentage of surviving cells depends on the dose level, and the level giving 50% inhibition was considered the IC50 dose level. The observed inhibition was dose-dependent, with a typical sigmoidal shape of dose-response, corroborating the validity of the observations. The IC50 of the EGCG against MDA-MB-231 was 83 µM/ml, indicating high cytotoxic activity.

### Analysis of microRNAs

**FIGURE 2:**
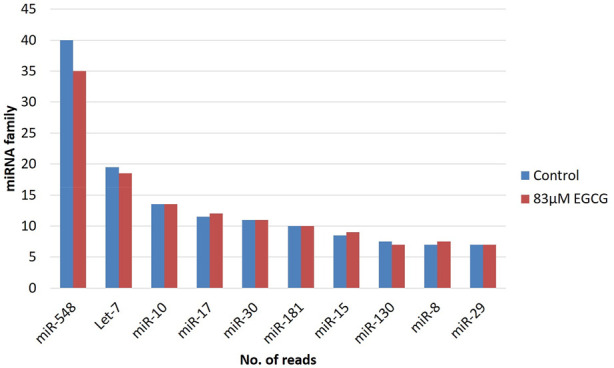
microRNA family distribution of known microRNAs in the untreated control and 24 h of 83 μM EGCG treatment. Color key-blue: untreated control, red: 83 μM EGCG treatment.

**FIGURE 3:**
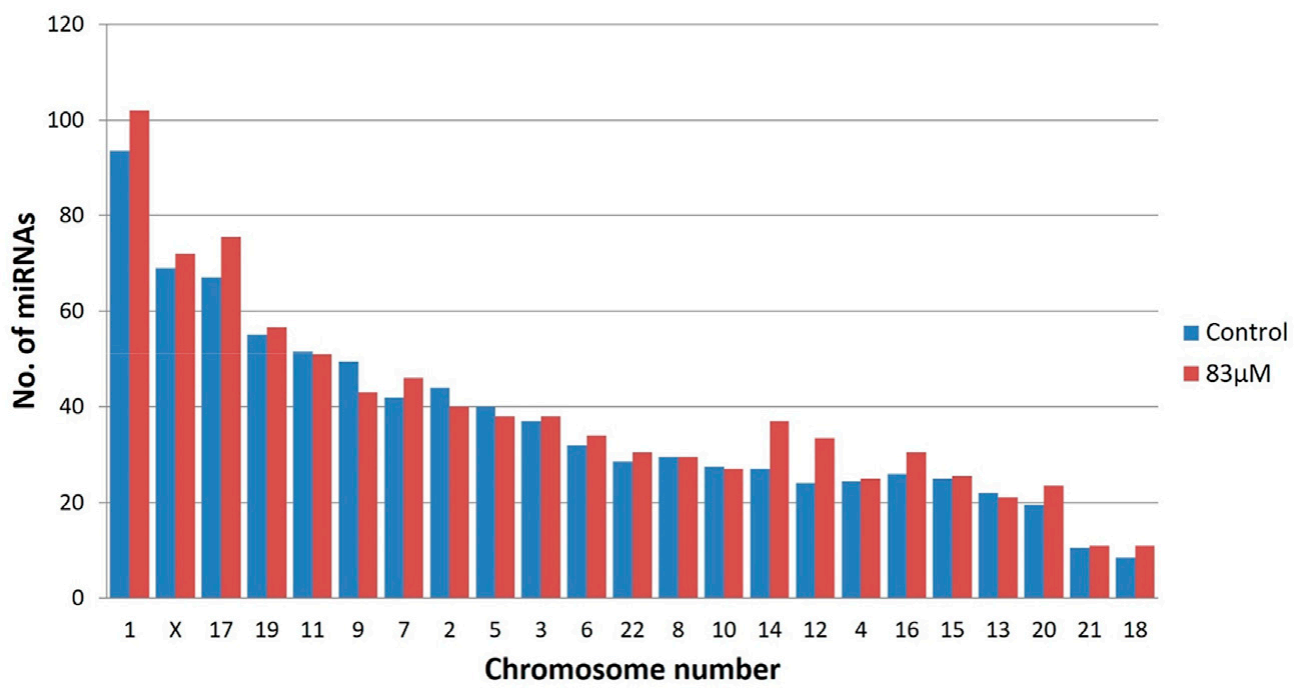
Chromosomal distribution of known microRNAs in the untreated control and 24 h of 83 μM EGCG treatment. Color key-blue: untreated control, red: 83 μM EGCG treatment.

The miRBase-21 database was used for known miRNA detection using the sequence similarity approach (ncbi-blast-2.2.30+). The novel miRNA sequences were predicted using MiReap_0.22b (Ji et al., 2013). In total, 875 and 960 known miRNAs were detected in the untreated control and 83 µM EGCG treatment respectively among which 59 and 82 miRNAs showed ≥50 read count, respectively. miRNA family analysis revealed the miR-548 family as the most abundant family followed by let-7, miR-10, miR-17, miR-30, miR-181, miR-15, miR-130, miR-8, and miR-29 family (Figure 2). In addition, on average, maximum miRNAs were predicted from chromosome 1 followed by chromosomes X and 17 (Figure 3). About 94 and 102 miRNAs were predicted from chromosome 1 followed by 69 and 72 miRNAs from chromosome X in the untreated control and 83 µM EGCG treatment, respectively.

### MicroRNA expression signature of MDA-MB-231

**FIGURE 4:**
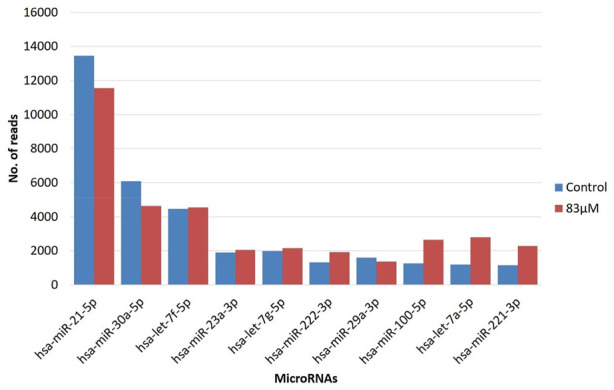
Read count of top ten highly expressed microRNAs in MDA-MB-231 cells in the untreated control vs. 24 h of 83 μM EGCG treatment. Color key-blue: untreated control, red: 83 μM EGCG treatment.

We identified top ten, highly expressed miRNAs (hsa-miR-21-5p, hsa-miR-30a-5p, hsa-let-7f-5p, hsa-miR-23a-3p, hsa-let-7g-5p, hsa-miR-222-3p, hsa-miR-29a-3p, hsa-miR-100-5p, hsa-let-7a-5p, hsa-miR-221-3p) in both the replicates of untreated control and 83 μM EGCG treatment by the integrated analysis (Figure 4). Hsa-miR-21-5p was significantly up-expressed in the untreated control and 83 μM EGCG treatment with the read count of 13461and 11,550 respectively, followed by hsa-miR-30a-5p with 6,080 and 4,639 read counts in untreated control and 83 μM EGCG treatment, respectively. Surprisingly, according to the NGS data, most of the highly expressed miRNAs showed consistent expression in the untreated control vs. 83 μM EGCG treatment.

### Prediction of putative novel microRNAs

**FIGURE 5:**
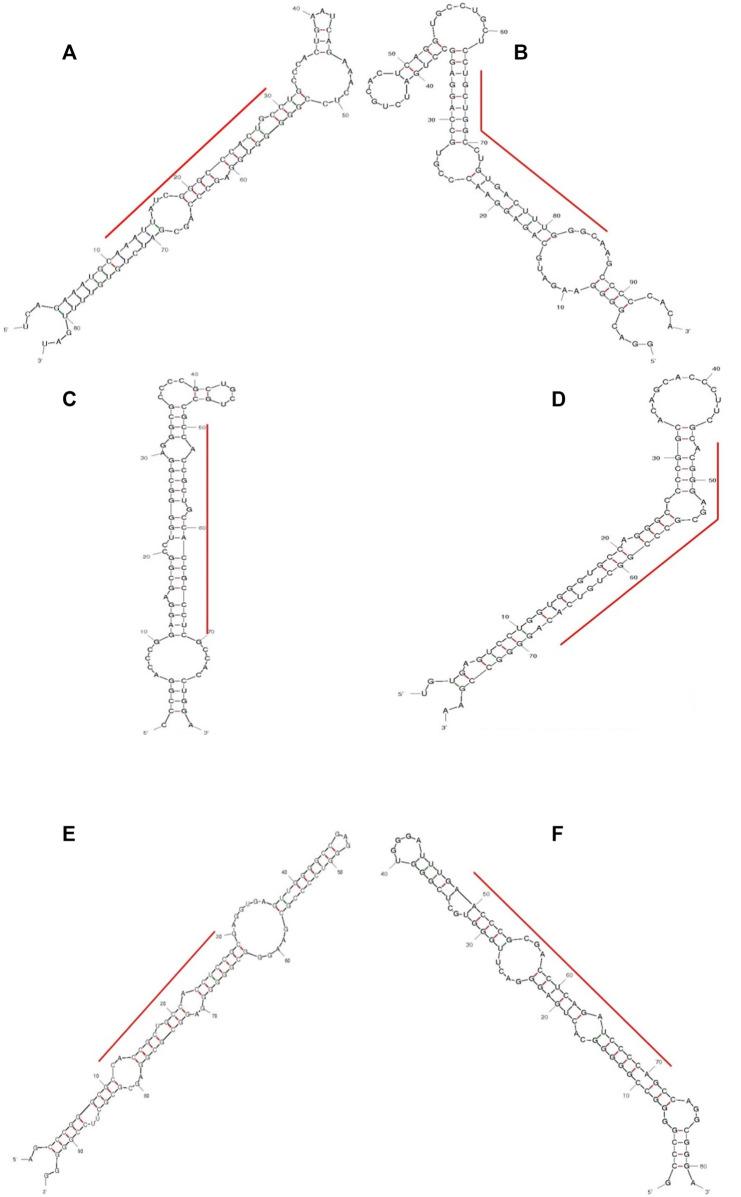
Predicted secondary structures of selected putative pri-miRNA sequences with high read counts from control **(A–C)** and 24 h of 83 μM EGCG treatment **(D–F)**. **(A)** EGCG-MDAMB231-1, **(B)** EGCG-MDAMB231-2, **(C)** EGCG-MDAMB231-3, **(D)** EGCG-MDAMB231-11, **(E)** EGCG-MDAMB231-12, **(F)** EGCG-MDAMB231-6.

All the novel miRNAs identified are given miRNA IDs e.g., EGCG-MDAMB231-1, EGCG-MDAMB231-2, and so on. We identified the top ten highly expressed putative novel miRNA sequences in control and 83 μM EGCG treatment. The miRNA IDs, miRNA sequences, chromosomal location, precursor, and mature sequences of the predicted putative novel miRNAs are presented in Tables 1, 2. Six putative novel miRNA sequences were expressed in control as well as treated samples. The predicted secondary structures of these putative novel miRNAs are presented in Figure 5. In the untreated control, the putative novel miRNA EGCG-MDAMB231-1 was highly expressed with a read count of 225 while in 83 μM EGCG treatment the read count was 173 (Table 1 A and B). Other than EGCG-MDAMB231-1, several other novel miRNAs are highly expressed as well as differentially expressed, e.g., EGCG-MDAMB231-4, EGCG-MDAMB231-5 showed prominent higher as well as differential expression.

**TABLE 1:** A: Top 10 putative microRNA sequences expressed in the untreated control. B: Top 10 putative microRNA sequences expressed in the 83 μM EGCG treatment.

MicroRNA ID	Sequence	Chromosome	Read count	Precursor start	Precursor end	Mature start	Mature end	Precursor sequence	MFE (Kcal/mol)
A									
EGCG-MDAMB231-1	AAA​TTA​TCG​GGC​CCA​CTG​CC	6	225	37155509	37155591	37155562	37155581	TCA​GAA​ATG​CAA​ATT​ATC​GGG​CCC​ACT​GCC​TGC​CCA​CTG​AAT​CAG​AAA​CTC​CGG​GGG​TGG​AGC​CCA​GCG​ATC​TGT​GTT​TTG​AT	−26.62
EGCG-MDAMB231-2	TGC​TGG​CCT​GTG​ACT​TTG​GGC​A	5	167	180370612	180370706	180370675	180370696	GGA​CGG​GGA​AGA​TGC​AGA​GGA​ACC​CGT​GCC​AGG​AGG​CCT​GAT​CTG​CAC​TCA​GGT​GCC​TGC​TCC​TGC​TGG​CCT​GTG​ACT​TTG​GGC​AAG​CCC​CCA​CA	−34.2
EGCG-MDAMB231-3	ACG​GGA​GCG​CCC​GGC​TGT​CAC	10	138	98268584	98268660	98268630	98268650	TGT​GAG​TCC​TGG​TGG​GTG​CCA​GGG​CCC​CCG​GCA​CAG​CAC​CCT​TCG​CAC​GGG​AGC​GCC​CGG​CTG​TCA​CAG​GGG​CCG​AA	−35
EGCG-MDAMB231-4	CAG​CAG​GGC​TGG​GTC​TTT​AT	3	124	9689747	9689818	9689789	9689808	GGG​CCA​TGG​GCA​GCA​GGG​CTG​GGT​CTT​TAT​GGA​GGG​CCT​GTG​GCA​TCC​AAA​TCA​CAC​CAG​CCT​ACT​GGT​CTT	−26.9
EGCG-MDAMB231-5	CTA​ACA​GCG​CCC​GGC​CTC​AG	12	114	122834713	122834785	122834723	122834742	CTG​TGC​CAG​CCT​AAC​AGC​GCC​CGG​CCT​CAG​CCC​CCA​TTG​TCC​CTG​GAG​CTG​GCG​AGG​TGT​CCG​GTT​GCG​GAG​C	−25.6
EGCG-MDAMB231-6	AAC​CCG​CGA​CCT​CAG​ATC​CCC​A	2	106	219522577	219522656	219522587	219522608	GCC​CGG​GGC​CGG​GGG​CAC​TGA​GGG​ACT​TGG​GTG​CTC​GGG​TGG​GAT​TTG​AAC​CCG​CGA​CCT​CAG​ATC​CCC​AGC​CAG​GCG​GG	−38.7
EGCG-MDAMB231-7	CCT​TTA​GCG​CCC​GGC​CGG​TCC	7	87	149624727	149624804	149624737	149624757	TGC​GTC​AAC​GCC​TTT​AGC​GCC​CGG​CCG​GTC​CGC​ACT​GTA​TCC​TGG​GAG​CCG​GCG​CGG​CCG​ACG​AAG​GCA​CAT​GAG​GCT	−37.4
EGCG-MDAMB231-8	ATC​CAG​CGC​CCG​GCC​TGG​CC	7	84	157554290	157554377	157554348	157554367	AGC​GGG​CAG​GAT​CCA​GCG​CCC​GGC​CTG​GCC​GCA​CCC​ATG​CCC​AGG​AGG​GCA​CGA​GAG​CGG​GCA​GGA​TCC​GGC​GCC​CGG​CCT​GGC​CGC​A	−56.6
EGCG-MDAMB231-9	CCA​AGT​ATC​GGG​CCC​AGC​TC	17	83	18093842	18093937	18093852	18093871	GCT​AAG​CTC​GCC​AAG​TAT​CGG​GCC​CAG​CTC​CTG​GAA​CCG​TCC​AAA​TCG​GCC​TCG​TCC​AAA​GGA​GAG​GGC​TTT​GAT​GTC​ATG​AAG​TCG​GGT​GAT​GCC	−30.9
EGCG-MDAMB231-10	TCA​GGA​GCG​CCC​GGC​CGT​CG	2	83	74529630	74529710	74529640	74529659	AAC​TGC​TGC​TTC​AGG​AGC​GCC​CGG​CCG​TCG​CCG​CCG​CCG​CCA​TTT​TCG​CGC​CCG​GCC​GCA​GGG​GCT​CTT​GGG​AAG​GCG​GAG	−38.32
B									
EGCG-MDAMB231-1	AAA​TTA​TCG​GGC​CCA​CTG​CCT	6	173	37155509	37155591	37155561	37155581	TCA​GAA​ATG​CAA​ATT​ATC​GGG​CCC​ACT​GCC​TGC​CCA​CTG​AAT​CAG​AAA​CTC​CGG​GGG​TGG​AGC​CCA​GCG​ATC​TGT​GTT​TTG​AT	−26.62
EGCG-MDAMB231-11	CCA​CCG​CTG​CCA​CCG​CCC​TC	1	116	226186538	226186616	226186548	226186567	CCC​GGA​CCC​GGA​GGA​GCG​GCC​TGG​GGC​GGA​GGG​CGC​CCC​GCT​GCT​GCC​GCC​ACC​GCT​GCC​ACC​GCC​CTC​GCC​ACC​TGG​A	−37.3
EGCG-MDAMB231-12	CCA​CCG​CTG​CCA​CCT​CCG​CG	12	82	124567246	124567339	124567310	124567329	AGC​CCG​GGC​GCC​ACC​GCT​GCC​ACC​TCC​GCG​AGG​TGA​GTT​GGG​GCC​GAG​GGT​CCC​CGC​GAA​GGG​CGG​GGG​GAG​GCG​CGG​AGC​GCG​CTT​CCG​GGG​G	−55.9
EGCG-MDAMB231-6	AAC​CCG​CGA​CCT​CAG​ATC​CCC​AG	2	71	219522576	219522656	219522586	219522608	GCC​CGG​GGC​CGG​GGG​CAC​TGA​GGG​ACT​TGG​GTG​CTC​GGG​TGG​GAT​TTG​AAC​CCG​CGA​CCT​CAG​ATC​CCC​AGC​CAG​GCG​GGA	−39.8
EGCG-MDAMB231-9	CCA​AGT​ATC​GGG​CCC​AGC​TC	17	64	18093842	18093937	18093852	18093871	GCT​AAG​CTC​GCC​AAG​TAT​CGG​GCC​CAG​CTC​CTG​GAA​CCG​TCC​AAA​TCG​GCC​TCG​TCC​AAA​GGA​GAG​GGC​TTT​GAT​GTC​ATG​AAG​TCG​GGT​GAT​GCC	−30.9
EGCG-MDAMB231-4	CAG​CAG​GGC​TGG​GTC​TTT​AT	3	49	9689747	9689818	9689789	9689808	GGG​CCA​TGG​GCA​GCA​GGG​CTG​GGT​CTT​TAT​GGA​GGG​CCT​GTG​GCA​TCC​AAA​TCA​CAC​CAG​CCT​ACT​GGT​CTT	−26.9
EGCG-MDAMB231-8	ATC​CAG​CGC​CCG​GCC​TGG​CC	7	39	157554290	157554377	157554348	157554367	AGC​GGG​CAG​GAT​CCA​GCG​CCC​GGC​CTG​GCC​GCA​CCC​ATG​CCC​AGG​AGG​GCA​CGA​GAG​CGG​GCA​GGA​TCC​GGC​GCC​CGG​CCT​GGC​CGC​A	−56.6
E-MDAMB231-13	AGG​GAG​GTC​CCT​GGT​GTC​TGG	12	37	122681826	122681897	122681867	122681887	TGG​TGG​CCA​CAG​GGA​GGT​CCC​TGG​TGT​CTG​GCT​GCA​TGC​TGG​CCA​TGG​TGA​CCA​GGT​GTC​CTT​GGG​CAG​GAG	−31.2
EGCG-MDAMB231-2	TGC​TGG​CCT​GTG​ACT​TTG​GGC​A	5	35	180370612	180370706	180370675	180370696	GGA​CGG​GGA​AGA​TGC​AGA​GGA​ACC​CGT​GCC​AGG​AGG​CCT​GAT​CTG​CAC​TCA​GGT​GCC​TGC​TCC​TGC​TGG​CCT​GTG​ACT​TTG​GGC​AAG​CCC​CCA​CA	−34.2
EGCG-MDAMB231-14	GGG​CAA​GGC​GTC​TGT​TTT​GCC	6	35	28944582	28944673	28944643	28944663	TAG​CGC​AGT​AGG​CAG​CGC​GTC​AGT​CTC​ATA​ATC​TGA​AGG​TCC​TGA​GTT​CGA​ACC​TCA​GAG​GGG​GCA​AGG​CGT​CTG​TTT​TGC​CAT​TTT​ACT​TC	−28.4

**TABLE 2:** A: List of all known up-regulated microRNAs after 83 μM EGCG treatments. B: List of all known down-regulated microRNAs after 83 μM EGCG treatments.

Treatments compared	No. of up-regulated microRNAs	Up-regulated microRNAs
A		
Control vs. 83μM EGCG treatment	106	hsa-miR-3135b, hsa-miR-214-3p, hsa-miR-7111-3p, hsa-miR-150-5p, hsa-miR-184, hsa-miR-365b-5p, hsa-miR-15a-3p, hsa-miR-363-3p, hsa-miR-6511a-3p, hsa-miR-155-5p, hsa-miR-365a-5p, hsa-miR-338-5p, hsa-miR-4284, hsa-miR-320a, hsa-miR-4436b-5p, hsa-miR-6806-3p, hsa-miR-6882-5p, hsa-miR-642a-5p, hsa-miR-5090, hsa-miR-548am-3p, hsa-miR-6769b-3p, hsa-miR-3605-3p, hsa-miR-4999-5p, hsa-miR-6811-5p, hsa-miR-4488, hsa-miR-6716-3p, hsa-let-7e-5p, hsa-miR-3184-3p, hsa-miR-1249-3p, hsa-miR-3138, hsa-miR-192-3p, hsa-miR-1908-3p, hsa-miR-7851-3p, hsa-miR-4781-3p, hsa-miR-1269a, hsa-miR-3120-3p, hsa-miR-6515-5p, hsa-miR-129-2-3p, hsa-miR-6851-3p, hsa-miR-574-5p, hsa-miR-193b-5p, hsa-miR-338-3p, hsa-miR-4661-5p, hsa-miR-676-3p, hsa-miR-4485-3p, hsa-miR-6511b-5p, hsa-miR-6786-3p, hsa-miR-6739-3p, hsa-miR-1293, hsa-miR-3150a-5p, hsa-miR-4707-5p, hsa-miR-199a-5p, hsa-miR-664b-3p, hsa-miR-3679-3p, hsa-miR-3145-3p, hsa-miR-122-5p, hsa-miR-4677-5p, hsa-miR-130a-5p, hsa-miR-411-5p, hsa-miR-548ab, hsa-miR-143-3p, hsa-miR-378a-3p, hsa-miR-550b-3p, hsa-miR-556-5p, hsa-miR-584-3p, hsa-miR-3177-3p, hsa-miR-324-5p, hsa-miR-320c, hsa-miR-3074-3p, hsa-miR-3909, hsa-miR-6854-3p, hsa-miR-4645-5p, hsa-miR-6514-5p, hsa-miR-3152-3p, hsa-miR-6753-5p, hsa-miR-6761-5p, hsa-miR-3648, hsa-let-7d-5p, hsa-miR-6729-3p, hsa-miR-1272, hsa-miR-382-3p, hsa-miR-30c-5p, hsa-miR-146a-5p, hsa-miR-4791, hsa-miR-500b-3p, hsa-miR-3620-5p, hsa-miR-3155b, hsa-miR-3140-3p, hsa-miR-1237-3p, hsa-miR-1273a, hsa-miR-5697, hsa-miR-3684, hsa-miR-1233-3p, hsa-miR-362-5p, hsa-miR-18a-3p, hsa-miR-491-3p, hsa-miR-328-3p, hsa-miR-215-5p, hsa-miR-378c, hsa-miR-145-5p, hsa-miR-6741-3p, hsa-miR-374b-5p, hsa-miR-4466, hsa-miR-146a-3p, hsa-miR-138-1-3p, hsa-miR-6516-3p
Treatments compared	No. of down-regulated MicroRNAs	Down-regulated microRNAs
B		
Control vs. 83μM EGCG treatment	114	hsa-miR-551b-5p, hsa-miR-197-5p, hsa-miR-1260b, hsa-miR-33a-3p, hsa-miR-3918, hsa-miR-1914-3p, hsa-miR-577, hsa-miR-3944-3p, hsa-miR-624-3p, hsa-miR-1972, hsa-miR-6735-5p, hsa-miR-3199, hsa-miR-522-3p, hsa-miR-1277-5p, hsa-miR-3163, hsa-miR-653-3p, hsa-miR-21-3p, hsa-miR-6513-5p, hsa-miR-3613-5p, hsa-miR-4420, hsa-miR-217, hsa-miR-6733-5p, hsa-miR-5008-3p, hsa-miR-296-3p, hsa-miR-3619-3p, hsa-miR-34b-5p, hsa-miR-3127-5p, hsa-miR-1976, hsa-miR-627-5p, hsa-miR-100-3p, hsa-miR-627-3p,hsa-miR-301b-5p, hsa-miR-27a-3p, hsa-miR-26b-5p, hsa-miR-181b-3p, hsa-miR-3074-5p, hsa-miR-762, hsa-miR-6856-3p, hsa-miR-1538, hsa-miR-5003-5p, hsa-miR-3140-5p, hsa-miR-508-3p, hsa-miR-4709-5p, hsa-miR-4684-5p, hsa-miR-7155-5p, hsa-miR-1273e, hsa-miR-19b-1-5p, hsa-miR-33a-5p, hsa-miR-335-5p, hsa-miR-3064-5p, hsa-miR-3690, hsa-miR-1273c, hsa-miR-218-1-3p, hsa-miR-5699-5p, hsa-miR-6854-5p, hsa-miR-6871-3p, hsa-miR-5001-3p, hsa-miR-4668-5p, hsa-miR-548u, hsa-miR-6799-3p, hsa-miR-17-3p, hsa-miR-5584-5p, hsa-miR-5584-3p, hsa-miR-3680-3p, hsa-miR-588, hsa-miR-6879-3p, hsa-miR-6726-3p, hsa-miR-548ac, hsa-miR-1284, hsa-miR-548p, hsa-miR-4517, hsa-miR-942-3p, hsa-miR-7110-3p, hsa-miR-636, hsa-miR-4429, hsa-let-7i-3p, hsa-miR-3149, hsa-miR-6750-3p, hsa-miR-140-5p, hsa-miR-301a-3p, hsa-miR-190a-5p, hsa-miR-6802-3p, hsa-miR-6783-5p, hsa-miR-3190-3p, hsa-miR-1273h-5p, hsa-miR-6814-5p, hsa-miR-5006-3p, hsa-miR-6858-3p, hsa-miR-708-5p, hsa-miR-6876-5p, hsa-miR-3191-3p, hsa-miR-378h, hsa-miR-6804-5p, hsa-miR-5196-3p, hsa-miR-570-5p, hsa-miR-4289, hsa-miR-6895-5p, hsa-miR-19b-3p, hsa-miR-27b-3p, hsa-miR-516a-5p, hsa-miR-450a-5p, hsa-miR-362-3p, hsa-miR-489-3p, hsa-miR-589-3p, hsa-miR-3529-3p, hsa-miR-99a-5p, hsa-miR-4454, hsa-miR-30c-2-3p, hsa-miR-6891-5p, hsa-miR-30d-3p, hsa-miR-6720-3p, hsa-miR-2355-3p, hsa-miR-369-3p, hsa-miR-597-3p

### Differential expression analysis of known microRNAs

**FIGURE 6:**
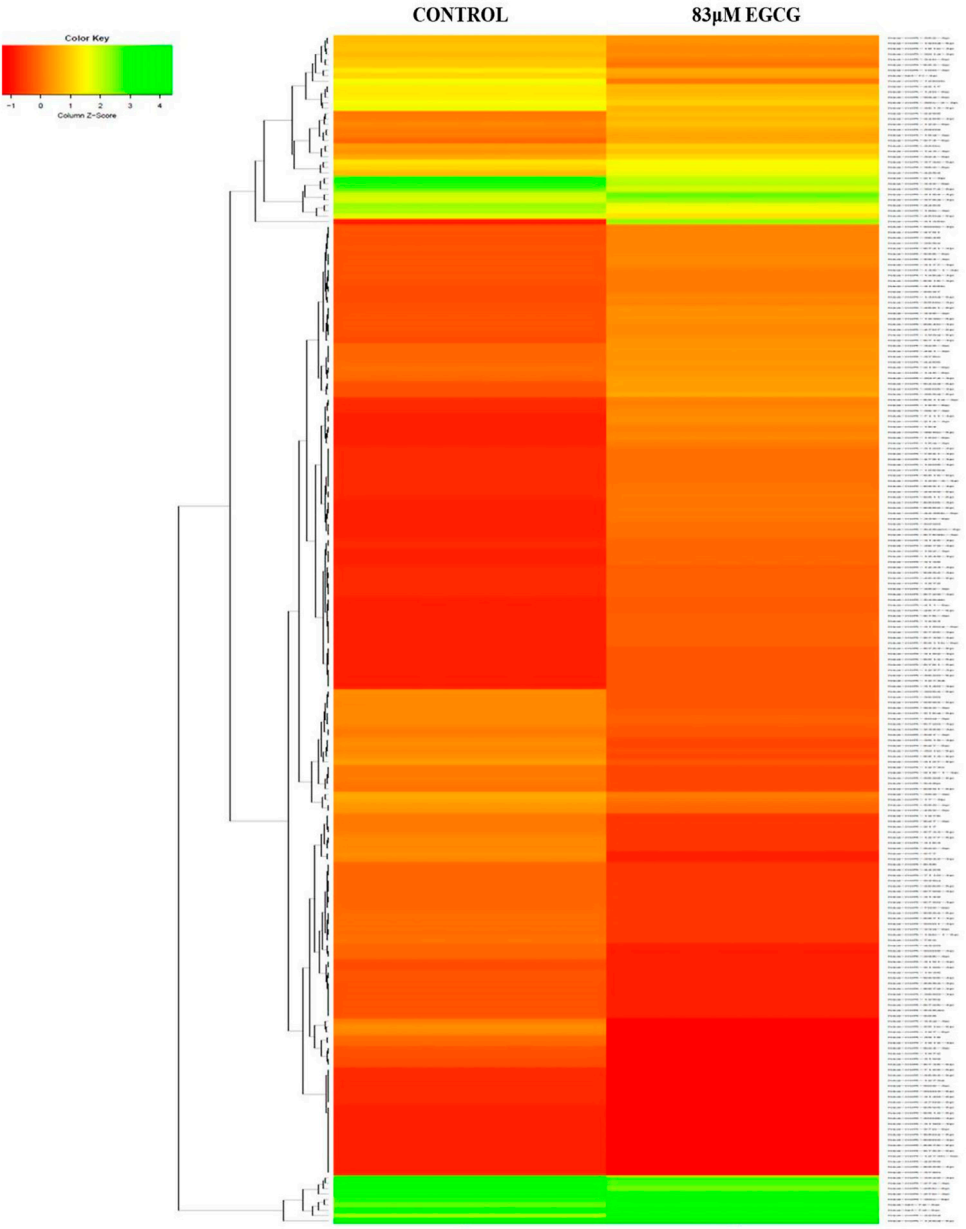
The expression profiles of known microRNAs in the untreated control MDA-MB-231 cells and 24 h of 83 μM EGCG treated cells. Color key-red: up-regulation, green: down-regulation, and yellow: neutral expression.

**FIGURE 7:**
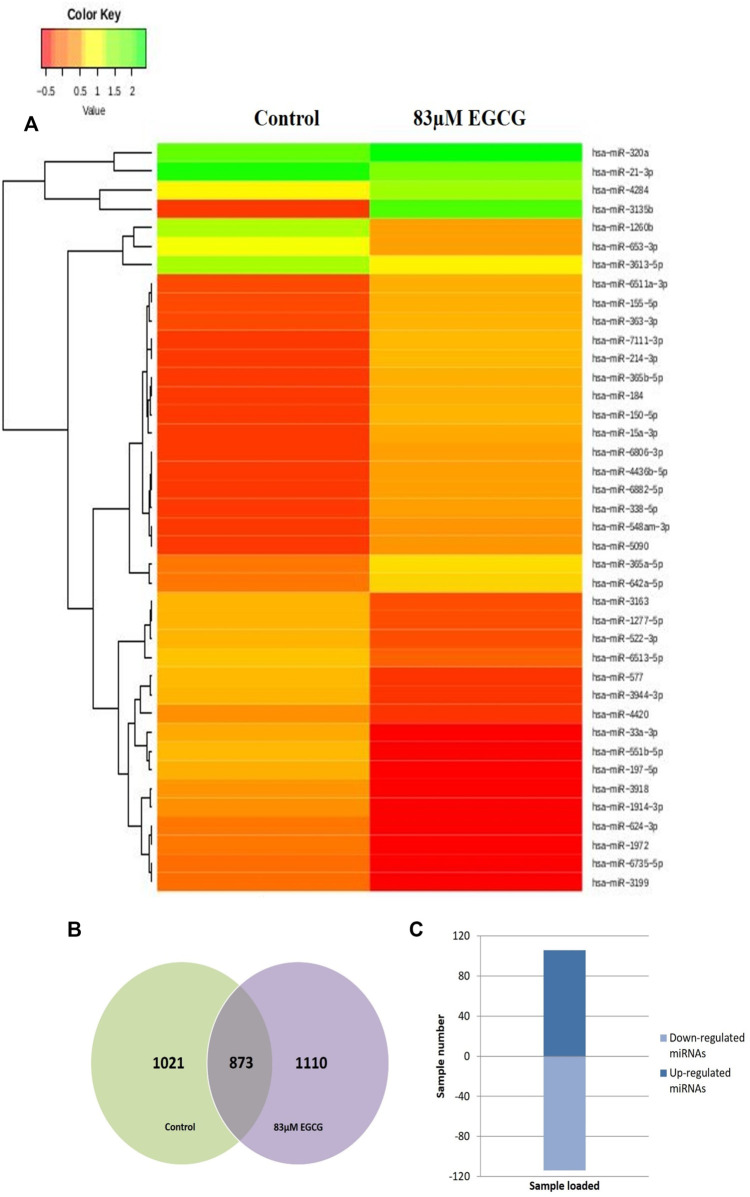
Dose-dependent known microRNA expression profile. **(A)** Relative microRNA expression levels of top 20 up- and down-regulated known microRNAs in the untreated control vs. 24 h of 83 µM EGCG treatment; Color key-red: up-regulation, green: down-regulation, and yellow: neutral expression. **(B)** Venn diagram is depicting the numbers of known microRNAs in untreated control and EGCG treated sample; **(C)** Summary set of up- and down-regulated known microRNAs exhibiting a change of expression after EGCG treatment.

**FIGURE 8:**
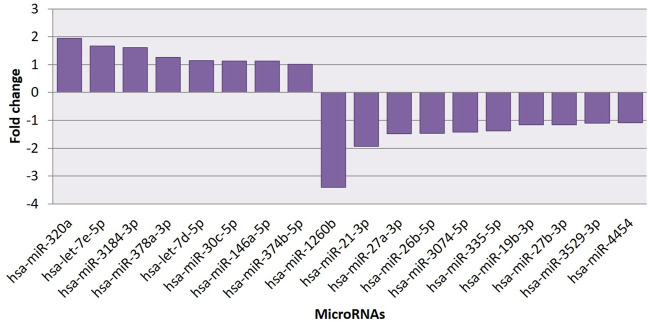
Common up- and down-regulated known microRNAs between untreated control vs 24 h of 83 μM EGCG treatments.

The effect of EGCG on MDA-MB-231 cells is indicated with a complete miRNA expression profile in Figure 6 miRNA expression with greater than 1.5 log2 fold change was determined in the untreated control vs. 83 μM EGCG treatment. Differential expressions of the top 20 up- and down-regulated miRNAs between the samples are presented in Figure 7A. A complete ID list of up- and down-regulated miRNAs is presented in Table 2. We observed a total expression of 1,021 known miRNAs in the untreated control sample, 1,110 known miRNAs in the 83 μM EGCG treated sample, and a total of 1,258 known miRNAs in both the samples (Figure 7B). Out of the 1,258 expressed miRNAs, 873 miRNAs were expressed in untreated control vs. 83 μM EGCG treated samples where 106 were up- and 114 were down-regulated (Figure 7C). By comparing the data with all the reported up-regulated miRNAs, hsa-miR-3135b showed the highest change of log2 fold expression in the untreated control vs. 83 μM EGCG treatment (7.26 log2 fold change). Furthermore, hsa-miR-551b-5p was highly down-regulated in the untreated control vs. 83 μM EGCG treatment (−3.64 log2 fold change). We observed 8 up- and 10 down-regulated miRNAs with high read count in the untreated control vs. 83 μM EGCG treatment (Figure 8).

### Differential expression analysis of putative novel microRNA sequences

**FIGURE 9:**
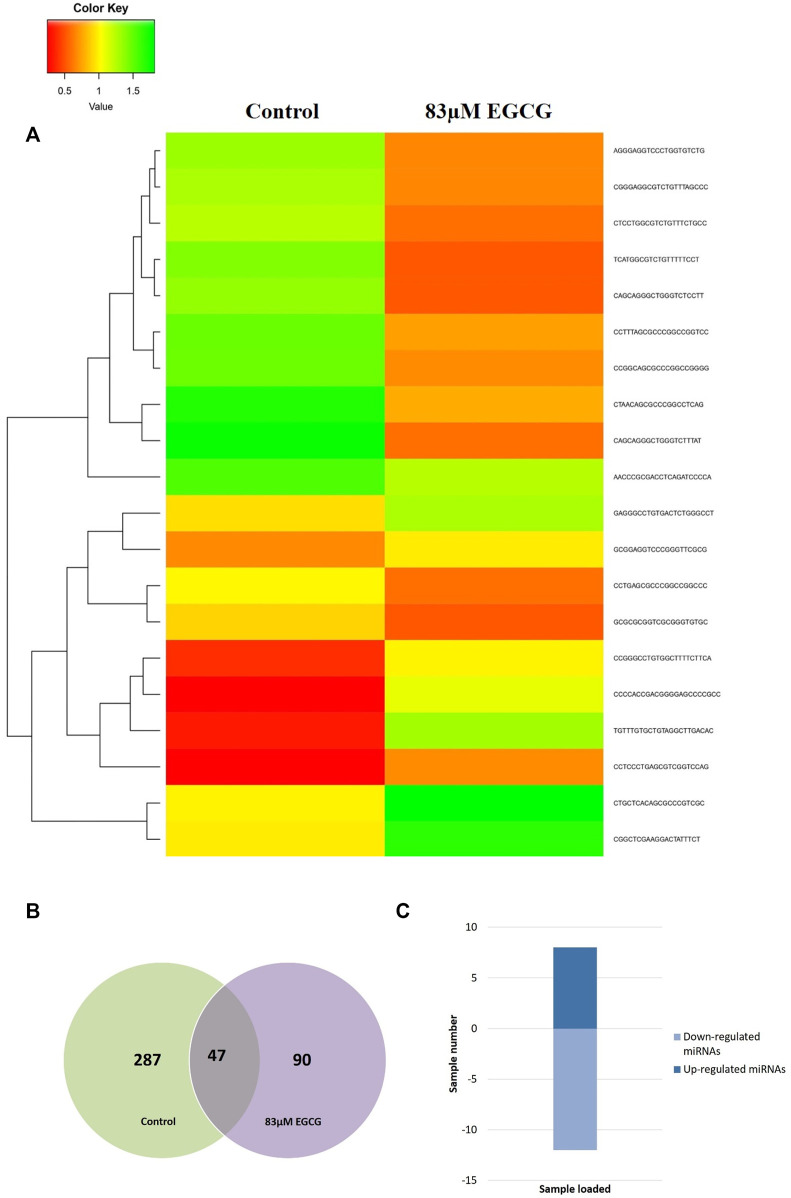
Dose-dependent expression profile of putative novel microRNAs. **(A)** Relative microRNA expression levels of top 20 up- and down-regulated novel microRNAs in untreated control vs. 24 h of 83 µM EGCG treatment; Color key-red: up-regulation, green: down-regulation, and yellow: neutral expression. **(B)** Venn diagram depicting the dose-dependent responses of novel microRNAs to EGCG; **(C)** Summary set of up- and down-regulated known microRNAs exhibiting a change of expression after EGCG treatments.

**FIGURE 10:**
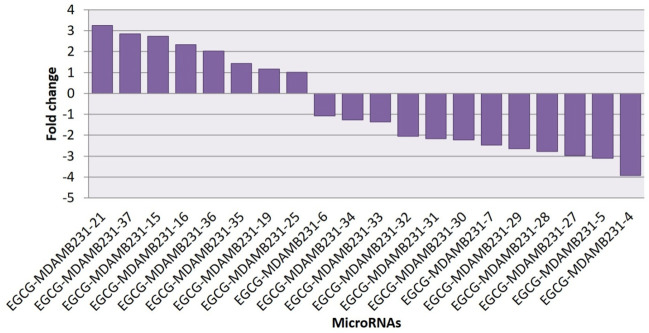
All up- and down-regulated novel microRNAs between untreated control vs. 24 h of 83 μM EGCG treatments.

Heatmaps were plotted to study the differential expression pattern of putative novel miRNAs. A complete putative miRNA profiling is shown in Figure 9A. We found 287 novel miRNAs expressed in the untreated control sample, 90 novel miRNAs expressed in the 83 μM EGCG treated sample, and a total of 377 expressed in both samples (Figure 9B). Out of the 377 expressed miRNAs, 47 were differentially expressed in the untreated control vs. 83 μM EGCG treatment, 8 were up- and 12 were down-regulated (Figure 9C). Differential expression of putative novel miRNAs in the untreated control vs. 83 μM EGCG treatment showing greater than 1.5 log2 fold change revealed 5 up-regulated and 9 down-regulated putative novel miRNAs in untreated control vs. 83 μM EGCG treatment. Among these putative novel miRNAs, EGCG-MDAMB231-21 was highly up-regulated and EGCG-MDAMB231-4 was the most down-regulated one (Table 3). The chromosomal location, precursor, and mature sequence details of putative novel miRNAs in the untreated control vs. 83 μM EGCG treatment are presented in Table 3. We observed 8 up- and 12 down-regulated putative novel miRNAs in the untreated control vs. 83 μM EGCG treatment (Figure 10).

**TABLE 3:** A: Up-expressed putative novel microRNA sequences after 83 μM EGCG treatment. B: Down-expressed putative novel microRNA sequences after 83 μM EGCG treatment.

Treatment compared	No. of microRNAs	MicroRNA ID	Log2fold change	Sequence	Chromosome	Precursor start	Precursor end	Mature start	Mature end	Precursor sequence	MFE (Kcal/mol)		
A											
Control vs. 83μM	8	EGCG-MDAMB231-21	3.25	TGT​TTG​TGC​TGT​AGG​CTT​GAC​AC	19	11577810	11577906	11577874	11577896	GGA​TCT​GGG​CAC​ACG​TGT​GCA​GCA​GCC​TCG​GCC​CAC​ACA​GCC​TCC​GGG​TGG​ACC​TGC​AGG​GGC​CTG​TTT​GTG​CTG​TAG​GCT​TGA​CAC​GTC​CAG​GTA​T	−44.4		
		EGCG-MDAMB231-37	2.84	CCC​CAC​CGA​CGG​GGA​GCC​CCG​CC	1	224340962	224341051	224340972	224340994	CCG​GCT​TCT​CCC​CCA​CCG​ACG​GGG​AGC​CCC​GCC​TGG​CCC​CAT​CGC​AGA​GAC​CCC​CAG​AGC​GGC​GGG​CTT​TTG​TTG​GGG​GGT​TCC​CGC​TTG	−37.5		
		EGCG-MDAMB231-15	2.73	CTG​CTC​ACA​GCG​CCC​GTC​GC	1	204997162	204997231	204997202	204997221	AGC​ATC​GGT​ACT​GCT​CAC​AGC​GCC​CGT​CGC​ACC​CAC​GGT​AGT​CGG​GGG​CAA​TGT​GGG​AGG​AGC​TGA​GAG​G	−28.8		
		EGCG-MDAMB231-16	2.32	CGG​CTC​GAA​GGA​CTA​TTT​CT	15	51483708	51483797	51483718	51483737	ACA​GGG​CCG​TGG​AAG​TGG​GCT​CTT​GGC​TGC​TTG​GCT​GGC​CTG​GTG​GTG​TGC​TGC​CAG​GGG​CGG​CTC​GAA​GGA​CTA​TTT​CTC​AGG​CGT​TGA	−42.51		
		EGCG-MDAMB231-36	2.01	CCG​GGC​CTG​TGG​CTT​TTC​TTC​A	16	51971845	51971931	51971900	51971921	AGA​TGG​GTA​ACC​GGG​CCT​GTG​GCT​TTT​CTT​CAG​CCA​ATG​AGG​AGG​AAG​AGA​GGG​TGG​GGG​AGG​GTG​ATA​GGG​TCT​GCA​TAT​CCA​TAT	−34.19		
		EGCG-MDAMB231-35	1.42	CCT​CCC​TGA​GCG​TCG​GTC​CAG	9	133452986	133453073	133452996	133453016	GGC​CCA​GTG​CCC​TCC​CTG​AGC​GTC​GGT​CCA​GAA​CGG​CAC​TCT​CGT​CCC​TCC​TGT​GAC​GCT​CTG​CTT​GGC​ACT​TTG​GGA​GGC​TGA​GGC​G	−36.22		
		EGCG-MDAMB231-19	1.17	GAG​GGC​CTG​TGA​CTC​TGG​GCC​T	3	49022514	49022599	49022568	49022589	CAA​GCA​GGG​TGA​GGG​CCT​GTG​ACT​CTG​GGC​CTC​AGT​TTC​CAC​ACT​GCG​GGC​CAG​GAC​TCA​CGT​TCC​ACT​GCA​CCA​CCG​AGT​GCG​GG	−34.6		
		EGCG-MDAMB231-25	1.006	GCG​GAG​GTC​CCG​GGT​TCG​CG	12	48351046	48351114	48351084	48351104	AGC​GGC​GGG​CGC​GGA​GGT​CCC​GGG​TTC​GCG​TTT​GGG​GGC​GCC​TGA​GCC​GCA​GCC​CCG​CCC​CCT​CCC​GTC	−34.1		
B												
Control vs. 83μM	12	EGCG-MDAMB231-4	−3.92	CAG​CAG​GGC​TGG​GTC​TTT​AT	3	9689747	9689818	9689789	9689808	GGG​CCA​TGG​GCA​GCA​GGG​CTG​GGT​CTT​TAT​GGA​GGG​CCT​GTG​GCA​TCC​AAA​TCA​CAC​CAG​CCT​ACT​GGT​CTT	−26.9		
		EGCG-MDAMB231-5	−3.09	CTA​ACA​GCG​CCC​GGC​CTC​AG	12	122834713	122834785	122834723	122834742	CTG​TGC​CAG​CCT​AAC​AGC​GCC​CGG​CCT​CAG​CCC​CCA​TTG​TCC​CTG​GAG​CTG​GCG​AGG​TGT​CCG​GTT​GCG​GAG​C	−25.6		
		EGCG-MDAMB231-27	−2.9738	TCA​TGG​CGT​CTG​TTT​TTC​CT	3	150147179	150147252	150147189	150147208	GCA​CAG​TGG​AAA​GGA​GAA​TGC​GTG​ACG​GCA​TTA​CAT​AGG​AAA​TGT​CAT​GGC​GTC​TGT​TTT​TCC​TCC​CAC​TTG​TG	−29.9		
		EGCG-MDAMB231-28	−2.78052	CAG​CAG​GGC​TGG​GTC​TCC​TT	21	34544707	34544781	34544752	34544771	TGC​TTC​CTG​ACA​GCA​GGG​CTG​GGT​CTC​CTT​TAC​TTT​CTA​TCT​TGG​GGA​CCA​GCA​GTC​TCC​TCT​GAC​AAG​GAA​GAG	−28.86		
		EGCG-MDAMB231-29	−2.64584	CCG​GCA​GCG​CCC​GGC​CGG​GG	14	100587445	100587522	100587493	100587512	CCC​GCC​AGC​CCC​GGC​CCC​GCG​CAG​CCG​CGG​CTG​AGC​CCG​CGC​GTC​CTC​CCG​GCA​GCG​CCC​GGC​CGG​GGC​GCA​AGT​GGT	−48.4		
		EGCG-MDAMB231-7	−2.47027	CCT​TTA​GCG​CCC​GGC​CGG​TCC	7	149624727	149624804	149624737	149624757	TGC​GTC​AAC​GCC​TTT​AGC​GCC​CGG​CCG​GTC​CGC​ACT​GTA​TCC​TGG​GAG​CCG​GCG​CGG​CCG​ACG​AAG​GCA​CAT​GAG​GCT	−37.4		
		EGCG-MDAMB231-30	−2.22583	AGG​GAG​GTC​CCT​GGT​GTC​TG	12	122681826	122681897	122681868	122681887	TGG​TGG​CCA​CAG​GGA​GGT​CCC​TGG​TGT​CTG​GCT​GCA​TGC​TGG​CCA​TGG​TGA​CCA​GGT​GTC​CTT​GGG​CAG​GAG	−31.2		
		EGCG-MDAMB231-31	−2.16401	CTC​CTG​GCG​TCT​GTT​TCT​GCC	13	112195054	112195151	112195121	112195141	ACA​GCA​GCC​CAC​CCC​AGG​CTC​CTG​GTG​CTG​TGA​AGG​CTG​ATT​CCG​ATT​CCC​AGG​AGG​TGG​GCA​GTG​TCT​CCT​GGC​GTC​TGT​TTC​TGC​CCC​TGC​TAA​TA	−35.8		
		EGCG-MDAMB231-32	−2.05245	CGG​GAG​GCG​TCT​GTT​TAG​CCC	8	127735191	127735285	127735255	127735275	GGG​CCC​CGT​GCG​GGA​GGC​GTC​TGT​TTA​GCC​CTG​AGA​TGT​GTC​TGC​CTG​TTC​CAG​AGC​TGG​GCT​AGG​GCG​AGA​GGG​AGG​TTG​CCT​GCT​CTC​TGC​CA	−39.7		
		EGCG-MDAMB231-33	−1.37073	CCT​GAG​CGC​CCG​GCC​GGC​CC	21	46458847	46458925	46458896	46458915	CCC​GCG​CGC​TCC​TGA​GCG​CCC​GGC​CGG​CCC​TAC​ACG​GGA​GCG​CGT​GCG​CGG​CGG​GAA​GGG​CGG​GTA​GCG​AGC​GCG​CGT​G	−50.3		
		EGCG-MDAMB231-34	−1.27119	GCG​CGC​GGT​CGC​GGG​TGT​GC	10	60944049	60944137	60944059	60944078	AGC​CAC​GGC​TGC​GCG​CGG​TCG​CGG​GTG​TGC​GGG​GCC​CCT​GCG​CGC​GGC​GCG​CCG​CCC​GCC​GCC​CAA​CTT​TGC​ACA​AAG​GCA​GCA​TGG​CA	−48.1		
		EGCG-MDAMB231-6	−1.07549	AAC​CCG​CGA​CCT​CAG​ATC​CCC​A	2	219522577	219522656	219522587	219522608	GCC​CGG​GGC​CGG​GGG​CAC​TGA​GGG​ACT​TGG​GTG​CTC​GGG​TGG​GAT​TTG​AAC​CCG​CGA​CCT​CAG​ATC​CCC​AGC​CAG​GCG​GG	−38.7		

### qRT-PCR analysis of microRNAs

**FIGURE 11:**
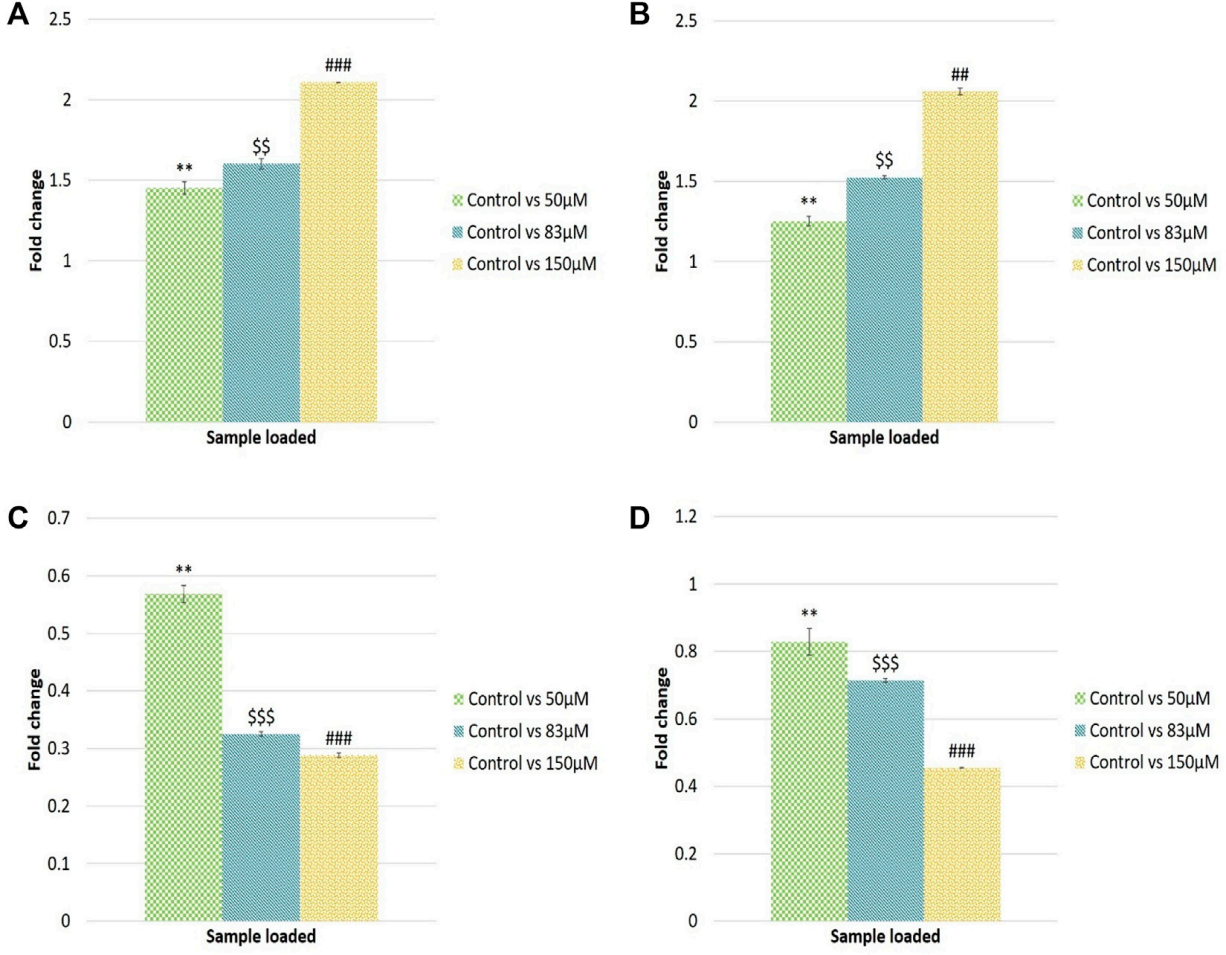
Validation of randomly selected known microRNAs by qRT-PCR. **(A)** Log2 fold change of hsa-let-7e-5p; **(B)** Log2 fold change of hsa-miR-320a; **(C)** Log2 fold change of hsa-miR-21-3p; **(D)** Log2fold change of hsa-miR-27a-3p microRNAs. The significance of differences between control vs. 50 µM EGCG indicated by “*”; control vs. 83 µM EGCG indicated by “$”; control vs. 150 µM EGCG indicated by “#”. Significance levels of *p* < 0.005 (“***”; “$$$”; “###”), *p* < 0.05 (“**”; “$$”; “##”) are denoted. EGCG treatments were given for 24 h. Data are presented as mean ± SD.

The qRT-PCR analysis was performed to validate the NGS dataset. Hsa-let-7e-5p, hsa-miR-320a, hsa-miR-21-3p and hsa-miR-27a-3p miRNAs were selected based on the read counts for the miRNA expression study. Furthermore, expression analysis of miRNAs in response to different EGCG treatments was studied. qRT-PCR analysis showed 1.45, 1.60 and 2.11- log2fold change of hsa-let-7e-5p expression in control vs. 50, 83, and 150 μM EGCG treatment. In the case of hsa-miR-320a, up-regulation of about 1.25, 1.52, and 2.05 log2fold change was observed for 50, 83, and 150 μM concentrations of EGCG treatment against an untreated control. A significant down-regulation of about 0.56, 0.32, and 0.28 log2fold was observed in the case of hsa-miR-21-3p, and 0.82, 0.71, and 0.45 log2fold were observed in the case of hsa-miR-27a-3p for 50, 83 and 150 μM EGCG treatments against the untreated control respectively (Figures 11A–D). In the miRNA sequencing data, a down-regulation of hsa-miR-21-3p by 1.93 log2 fold was observed in the untreated control vs. 83 μM EGCG treatment. Furthermore, a significant down-regulation of hsa-miR-27a-3p by 1.48 log2 fold change was noted in the untreated control vs. 83 μM EGCG treatment. About 1.95 log2 fold up-regulation of hsa-miR-320a was observed in the untreated control vs. 83 μM EGCG treatment. In hsa-let-7e-5p, the log2 fold change of 1.66 was noted in the untreated control vs. 83 μM EGCG treatment. A comparative fold change between the qRT-PCR and sequencing dataset supports each other. Therefore, q-RT PCR results validated the present NGS dataset.

**FIGURE 12:**
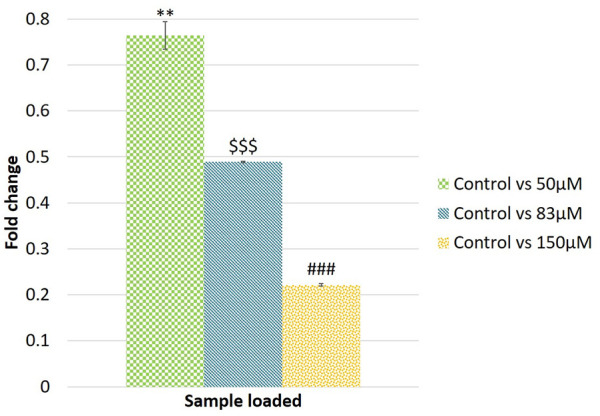
Validation of randomly selected known microRNAs by qRT-PCR. Log2 fold change of EGCG-MDAMB231-4. The significance of differences between control vs 50 µM EGCG indicated by “*”; control vs. 83 µM EGCG indicated by “$”; control vs. 150 µM EGCG indicated by “#”. Significance levels of *p* < 0.005 (“***”; “$$$”; “###”), *p* < 0.05 (“**”; “$$”; “##”) are denoted. EGCG treatments were given for 24 h. Data are presented as mean ± SD.

A qRT-PCR analysis of the putative novel miRNA EGCG-MDAMB231-4 showed 0.76, 0.48, and 0.22-fold change for 50, 83, and 150 μM EGCG treatment against untreated control (Figure 12). In the same way, the fold change was compared with NGS data. Log2 fold change obtained in the sequencing dataset gives support to qRT-PCR analysis. This qRT-PCR analysis also attests to our computational analysis of NGS data.

### KEGG and PANTHER pathway enrichment of targets of validated microRNAs

**FIGURE 13:**
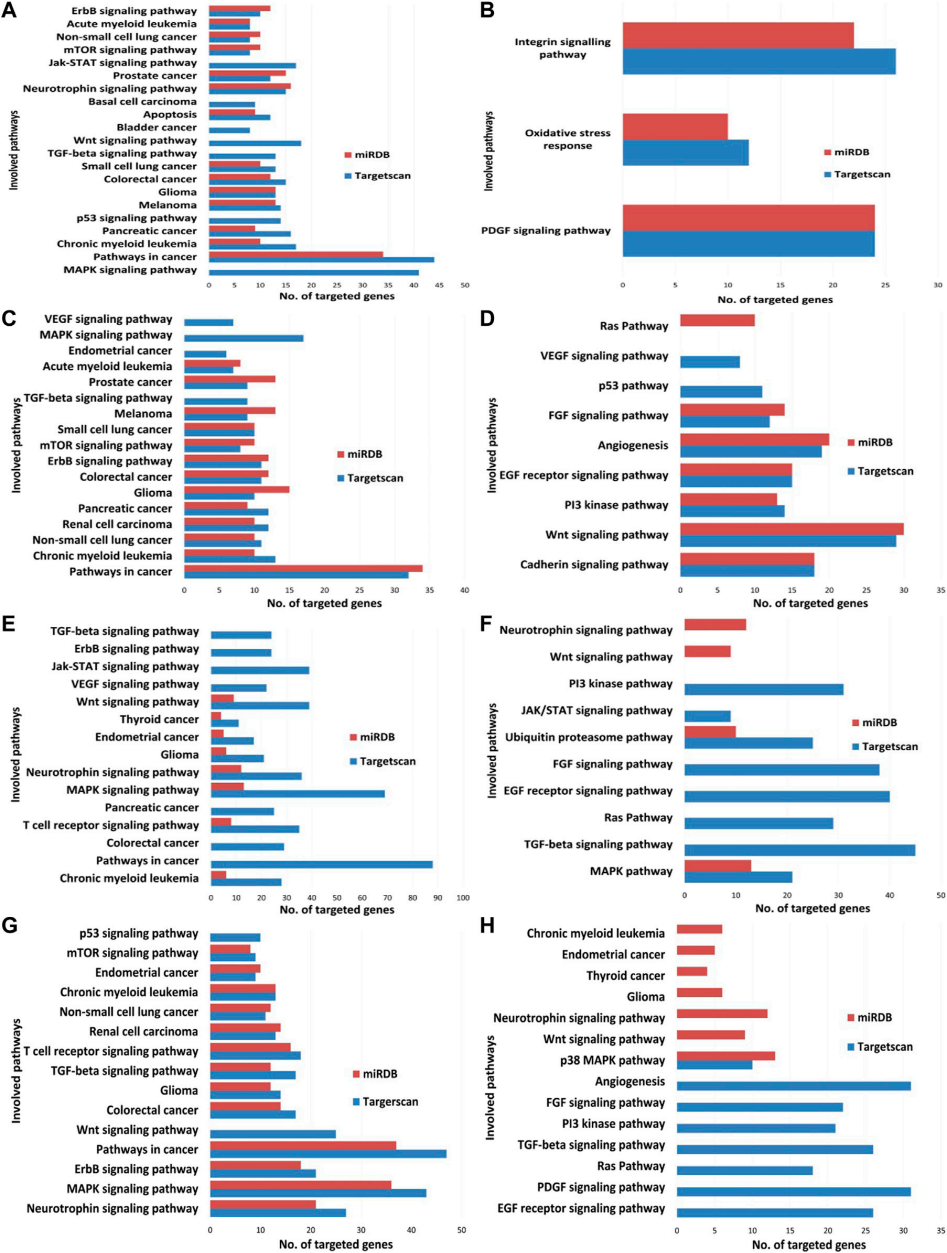
microRNA target validation enrichment data from KEGG and PANTHER database. **(A)** Analysis of has-let7e-5p from KEGG pathway database; **(B)** analysis of has-let7e-5p from PANTHER pathway database; **(C)** analysis of has-miR320a from KEGG pathway database; **(D)** analysis of has-miR320a from PANTHER pathway database; **(E)** analysis of has-miR21-3p from KEGG pathway database; **(F)** analysis of has-miR21-3p from PANTHER pathway database; **(G)** analysis of has-miR27a-3p from KEGG pathway database; **(H)** analysis of has-miR27a-3p from PANTHER pathway database.

The high precision target prediction for hsa-miR-21-3p, hsa-miR-27a-3p, hsa-let-7e-5p, and hsa-miR-320a was carried out using computational target prediction software TargetScan and miRDB. Default cut-off values were used for gene target prediction. KEGG and PANTHER pathway analysis were carried out using the Database for Annotation, Visualization, and Integrated Discovery (DAVID), and the pathways were shortlisted. Pathway analysis for miRNAs was evaluated and common pathways predicted between TargetScan and miRDB data are presented in Figure 13. Wnt, MAPK, mTOR, p53 pathway, pathways in cancer, regulation of actin cytoskeleton, jak-STAT pathway, ErbB signaling pathway, insulin signaling pathway, and axon guidance were a few significant pathways obtained in KEGG pathway analysis (Figures 13A,C,E,G). In addition, cadherin pathway, Wnt pathway, PI3K pathway angiogenesis, EGF signaling pathway, PDGF (Platelet-derived growth factor) signaling pathway, and oxidative stress response genes were reported in PANTHER pathway analysis (Figures 13B,D,F,H).

Furthermore, the Wnt signaling pathway as well as the MAPK pathway were observed one of the common and highly targeted pathways for hsa-miR-21-3p, hsa-miR-27a-3p, hsa-let-7e-5p, and hsa-miR-320a through KEGG pathway analysis (Table 4) but no common pathway was observed among the miRNAs from our analysis with PANTHER pathway. Wnt pathway, MAPK pathway, ErbB pathway, TGF-beta pathway, mTOR pathway, p53, PI3K pathway, and EGF (epidermal growth factor) receptor signaling pathway were the most significant pathways predicted by TargetScan and miRDB target list.

**TABLE 4:** List of common pathways in TargetScan and miRDB for two upregulated and two downregulated microRNA from NGS study.

microRNAs	KEGG pathways common in TargetScan and miRDB	PANTHER pathways common in TargetScan and miRDB	KEGG pathways common in all the microRNAs	PANTHER pathways common in all the microRNAs
Hsa-miR-21-3p	Wnt signaling pathway	MAPK signaling pathway	Wnt signaling pathway MAPK signaling pathway	No
	MAPK signaling pathway	TGF-beta signaling pathway		
	Ubiquitin mediated proteolysis	Neurotrophin signaling pathway		
	Neurotrophin signaling pathway	Wnt signaling pathway		
	GnRH signaling pathway			
hsa-miR-27a-3p	ErbB signaling pathway	EGF receptor signaling pathway		
	MAPK signaling pathway	PDGF signaling pathway		
	Wnt signaling pathway	Ras Pathway		
	Insulin signaling pathway	Insulin/IGF pathway-protein kinase B signaling cascade		
	TGF-beta signaling pathway			
hsa-let-7e-5p	P53 pathway	Oxidative stress response		
	Wnt signaling pathway	PDGF signaling pathway		
	MAPK signaling pathway	Integrin signaling pathway		
	Jak-STAT signaling pathway			
	Insulin signaling pathway			
	Adipocytokine signaling pathway			
hsa-miR-320a	mTOR signaling pathway	Cadherin signaling pathway		
	Pathways in cancer	Wnt signaling pathway		
	ErbB signaling pathway	PDGF signaling pathway		
	Insulin signaling pathway	PI3 kinase pathway		
	Wnt signaling pathway	Axon guidance mediated by netrin		
	MAPK signaling pathway			

For the prediction of the targeted gene and pathway analysis of novel miRNA, MiRanda software was used. The analysis suggested a few common cancer-related pathways like Wnt, angiogenesis, TGF-beta, p-53, PI3K, and p-38 MAPK, Notch, EGFR, and NF-κB pathways. The putative novel miRNA EGCG-MDAMB231-4 targets INHA (Inhibin Subunit Alpha), ERCC (ERCC excision repair 1), AREL1 (Apoptosis Resistant E3 Ubiquitin Protein Ligase 1) genes. Furthermore, the putative novel miRNA EGCG-MDAMB231-5 targets MBP (Myelin Basic Protein), HIC1 (hypermethylated in cancer 1), and P3H2 (Prolyl 3-Hydroxylase 2), SLC6A2 (Solute Carrier Family 6 Member 2) genes. In addition, RFC5 (Replication Factor C Subunit 5), CBLB (E3 ubiquitin-protein ligase CBL-B), DUSP7 (Dual Specificity Phosphatase 7) SMG7 (Nonsense Mediated mRNA Decay Factor), CSNK1G2 (Casein Kinase 1 Gamma 2), were targeted by EGCG-MDAMB231-27. Furthermore, TBC1D22A (TBC1 Domain Family Member 22A), GPI (Glucose-6-phosphate isomerase), ZNF527 (Zinc Finger Protein 527), KDM5C (Lysine Demethylase 5C), PRKCH (Protein kinase C eta type), SLC2A6 (Solute Carrier Family 2 Member 6) genes were targeted by EGCG-MDAMB231-6. The analysis of putative novel miRNA revealed to modulate cell cycle progression, TGF-beta pathway, Wnt pathway, gonadotropin-releasing hormone receptor pathway, nucleotide excision repair, EGFR pathway, MAPK pathway, etc. We believe that these predicted putative novel miRNA sequences play a major role in cancer proliferation and metastasis.

## Discussion

NGS analysis of miRNA expression change in EGCG-induced breast cancer cells was done using *in vitro* human breast cancer cell line MDA-MB-231.

It is evident from different scientific studies that tea polyphenol has anti-cancerous activity, especially EGCG, and it has also been studied against a variety of diseases as a potent therapeutic agent (Khan et al., 2006). EGCG acts against cancer in a multiple-way like anti-oxidation and inhibition of cell signaling pathways which causes cell proliferation, angiogenesis, metastasis, acceleration of apoptotic pathways, etc. (Kurahashi et al., 2008). EGCG has a cytotoxic effect on breast cancer by inhibiting its tumorigenesis independently of its ER status (Thangapazham et al., 2007). We found that EGCG can reduce the viability of MDA-MB-231 cells in a dose-dependent manner with 83 µM concentration as the IC50 value. EGCG has a high anti-cancer activity which can catalyze chemotherapy-induced apoptosis in breast cancer cell lines (Roy et al., 2005).

Our NGS study revealed 877 known miRNAs which were already reported to miRBase22.1 and 112 putative novel miRNAs expressed in breast cancer cell line MDA-MB-231. Among known and novel miRNAs, 654 known and 26 novel miRNAs exhibited no significant expression variation after EGCG treatment when compared to untreated control from which we can state that EGCG did not influence these miRNAs in MDA-MB-231 cells, and some known and novel miRNAs showed a drastic change in their expression after EGCG treatment which are mentioned in Figures 8, 10, respectively. Hence, the study of miRNA profile modulated by EGCG can be done using three independent criteria i.e., log2 fold expression study, differentially expressed up- and down-regulated miRNAs, and putative gene targets of the miRNAs.

In this study, more than 1.5 log2 fold change of miRNA expression was taken into account for the criteria of both known and putative novel miRNA analysis. Among 874 known miRNAs expressed in both untreated control cells and treated cells, about 106 (12.12%) were up-regulated and 114 (13.04%) were down-regulated after 83 µM EGCG treatment. Among 106 up-regulated miRNAs 39 (36.79% of up-expressed miRNA) and among 114 down-expressed miRNAs 32 (28.07% of down-expressed miRNA) showed greater than 1.5 log2 fold differential expression. In the case of putative novel miRNAs, 47 out of 112 miRNAs were expressed in both untreated control cells and treated cells. Among these 47 putative novel miRNAs expressed in both untreated control cells and treated cells about 8 (17.02%) were up-regulated and 12 (25.53%) were down-regulated after 83 µM EGCG treatment. Among 8 up-expressed miRNAs, 6 (75% of up-expressed miRNA) and among 12 down-expressed miRNA 8 (75% of down-expressed miRNA) showed greater than 1.5 log2 fold differential expression. From the present study, hsa-miR-3135b surprisingly showed a higher differential expression of about 7.26 log2 fold and in the case of putative novel miRNAs, EGCG-MDAMB231-4 showed a higher differential expression of about 3.92-log2fold. Limited studies have been conducted on miR-3135b and its role in different types of cancers is unknown, although it was up-regulated in tamoxifen resistant MCF-7 breast cancer cells (Chu et al., 2015). From this study, it is evident that hsa-miR-3135b is very much associated with breast cancer progression and EGCG is capable of modulating miRNA expression which can further modulate different cell signaling pathways related to cancer.

This NGS study revealed an interesting fact about highly expressed miRNAs. Top ten highly expressed miRNAs like hsa-miR-21-5p, hsa-let-7f-5p, hsa-miR-30a-5p, hsa-miR-23a-3p, hsa-let-7g-5p, hsa-miR-222-3p, hsa-miR-29a-3p, hsa-miR-100-5p, hsa-let-7a-5p, hsa-miR-221-3p, did not show any significant expression difference between untreated control and 83 µM EGCG treated cell of MDA-MB-231 (Figure 4). The most up-expressed miRNA among these ten is hsa-miR-21-5p which is a very common and frequently observed miRNA in different types of cancer and it became the first “oncomiR” for its oncogenic effect (Cho, 2007). Although has-miR-21-3p showed significant expression difference between control and treated samples in a down-regulatory manner. In breast cancer, a higher expression level of miR-21 is associated with poor prognosis, support cell proliferation, and invasion (Dong et al., 2014; Fang et al., 2017). miR-21 is associated with chemoresistance in breast cancer (Yu X. et al., 2016). Inhibition of miR-21 reduces the progression of breast cancer by targeting one of the well-known stem cell markers CD133 (Yin et al., 2019). Although most of the roles of miR-21 are attributed to has-miR-21-5p, the oncogenic role of hsa-miR-21-3p is also well established (Amirfallah et al., 2021). Our present study also showed differential expression of has-miR-21-3p after EGCG treatment. Therefore, we can hypothesize that EGCG treatment has a significant effect on hsa-miR-21-3p which can cause apoptosis of breast cancer cells.

Even though let7 is a well-known and well-studied miRNA family, hsa-let-7f-5p which is the second most highly expressed miRNA between control and EGCG treated samples has not been studied thoroughly against various cancers. Hsa-let-7f-5p has been found to repress various pro-apoptotic proteins and induce chemoresistance in colorectal cancer (Tie et al., 2018). Another highly expressed miRNA is miR-30a-5p known to negatively regulate cell growth, migration, invasion, and metastasis, autophagy in chronic myelogenous leukemia, and TGF-b1-induced epithelial-mesenchymal transition in cancer (Jiang L. H. et al., 2018). In the case of breast cancer, miR-30a suppresses cell growth, invasion, and metastasis by targeting *ROR1*, and loss of miR-30a expression causes oncogenesis (Wang et al., 2018). In our NGS data, miR-30a did not show any significant difference in expression between untreated control cells and EGCG-treated cells. Thus, it appears that EGCG does not affect the expression of miR-30a in MDA-MB-231 breast cancer cells. High expression of a tumor suppressor miRNA like miR-30a in untreated MDA-MB-231 breast cancer cells can portray that, breast cancer cells can compensate and resist the effect of miR-30a.

Differential analysis revealed that miR-27a-3p was one of the highly down-regulated miRNAs in response to EGCG treatment (Figure 8). Various experimental studies showed that miR-27a elicits both oncogenic and tumor suppressor roles in different cancers. It acts as a tumor suppressor in non-small cell lung cancer (NSCLC) by targeting the *HOXB8* gene (Yan et al., 2019). On the other hand, miR-27a promotes cell proliferation and suppresses apoptosis in colorectal cancer (CRC) by modulating *BTG1* (Su et al., 2019). In the case of breast cancer, miR-27a exhibits oncogenic characteristics by inducing epithelial-to-mesenchymal transition (EMT) (Jiang G. et al., 2018). The present study also revealed that miR-27a expression can be modulated by EGCG treatment which further caused a reduction in cancer progression.

The differential analysis also revealed upregulated miRNAs, among which let7e-3p and miR-320a were notable. Let-7e belongs to the miRNA family of let-7 known as a tumor suppressor miRNA (Figure 8). In the case of NSCLC, low expression of let-7 is associated with poor postoperative survival (Takamizawa et al., 2004). Moreover, the experimental study revealed that let-7 suppresses breast cancer cell migration and invasion *via* down-regulation of the C-C chemokine receptor 7 gene (Kim et al., 2012). miR-320a also acts as a tumor suppressor in various cancers. Lower expression of miR-320a is found in cervical cancer which generally inhibits cancer progression by regulating Mcl-1 (Zhang et al., 2016). In the case of breast cancer, miR320a inhibits breast cancer metastasis *via* suppression of a notable oncogene Metadherin (*MTDH*, Yu J. et al., 2016). The up-regulation of let-7e and miR-320a after EGCG treatment to breast cancer cells gave evidence that EGCG can help increase the expression of the two significant miRNAs which cause a decrease in cell growth and migration. To validate the differential expression data of known miRNAs, qRT-PCR was performed by choosing two up-regulated and two down-regulated miRNAs such as hsa-let-7e-5p, hsa-miR-320a, hsa-miR-21-3p, and hsa-miR-27a-3p. The NGS dataset and validation of the known miRNA with qRT-PCR confirmed the significant expression of these miRNAs (Figure 11).

The further goal of this study was to identify putative novel miRNA sequences in MDA-MB-231 cells and their potential differences in expression between untreated control and EGCG treatment. From the NGS analysis, a total of 47 putative novel miRNAs were reported within the untreated control and EGCG treated cells among which 8 were up-regulated and 12 were down-regulated (Table 3). To validate the differential expression data of novel miRNAs, qRT-PCR was performed. The NGS dataset and validation of the novel miRNA EGCG-MDAMB231-4 with qRT-PCR confirmed the significant expression of this putative novel miRNA (Figure 12). Further validation of the sequence is required.

Bioinformatics analysis of miRNAs with TargetScan and miRDB database revealed an average of 1,500 possible targeted genes. With the help of DAVID web-based gene ontology and pathway prediction software, potential gene ontology and pathway analysis were done using targeted genes from TargetScan and miRDB. KEGG pathway and PANTHER pathway inquiry were done. Target prediction and pathway analysis of hsa-miR-21-3p, hsa-miR-27a-3p, hsa-let-7e-5p, and hsa-miR-320a showed notable pathways like Wnt, MAPK, TGF-beta, Ras, p53, JAK-STAT, PI3K were being targeted (Figure 13). Among these, Wnt and MAPK pathways were commonly targeted by all four selected known miRNAs. Significant expression of these miRNAs in untreated control and EGCG-treated MDA-MB-231 cells demonstrated their important role in regulating cell proliferation. For target prediction of the putative novel miRNAs, on average, 130 target genes were predicted for each of them using MiRanda 3.3a. EGCG-MDAMB231-4, EGCG-MDAMB231-5, EGCG-MDAMB231-6, and EGCG-MDAMB231-27, were a few common putative novel miRNAs that were expressed differentially between untreated control cells and EGCG treated cells. These novel miRNAs target *INHA* (Inhibin Subunit Alpha), *ERCC* (ERCC excision repair 1), *AREL1* (Apoptosis Resistant E3 Ubiquitin Protein Ligase 1), *MBP* (Myelin Basic Protein), *HIC1* (hypermethylated in cancer 1), *P3H2* (Prolyl 3-Hydroxylase 2), *SLC6A2* (Solute Carrier Family 6 Member 2), *RFC5* (Replication Factor C Subunit 5), *CBLB* (E3 ubiquitin-protein ligase CBL-B), *GPI* (Glucose-6-phosphate isomerase), *ZNF527* (Zinc Finger Protein 527), *KDM5C* (Lysine Demethylase 5C), *PRKCH* (Protein kinase C eta type), *SLC2A6* (Solute Carrier Family 2 Member 6), etc. genes and regulate cell cycle progression, TGF-beta pathway, Wnt pathway, gonadotropin-releasing hormone receptor pathway, nucleotide excision repair, EGFR pathway, MAPK pathway, etc.

From this study, few notable miRNAs (e.g., has-miR-146a-5p, hsa-miR-425-5p, and hsa-miR-23b-3p) having significantly higher as well as differential expression in the cancer control and EGCG treated samples were identified which can be suitable for diagnostic and prognostic markers. One of the notable miRNAs found from this NGS study is has-miR-146a-5p. This miRNA affects cell proliferation and mutation in breast cancer by directly targeting the BRCA1 (Gao et al., 2018). Another prominent miRNA is hsa-miR-425-5p which was found to promote breast carcinogenesis by inducing PI3K/AKT pathway when it binds and phosphorylates PI3K, p58, AKT. It was also found to bind and suppress PTEN (Zhang et al., 2020). Hsa-miR-23b-3p is another miRNA with distinguished expression among the samples. Although its role has been studied among different cancer, no study has been done on its role in breast carcinoma. One research has been conducted evaluating the role of miR-23b along with miR-27b in breast cancer by knocking down these miRNAs using CRISPR/CAS9 which does not convey the individual role of miR-23b in the desired cancer type (Hannafon et al., 2019).

## Conclusion

EGCG was proven to be a potent anti-cancer compound against MDA-MB-231 breast cancer cell with an IC50 concentration of 83 µM miRNA profiling among cancer control and EGCG treated cells revealed 1,258 known and 330 unknown novel miRNAs in MDA-MB-231 breast cancer cell. Highly expressed miRNAs were both oncogenic and tumor suppressor miRNAs which infer breast cancer as a non-resistance yet aggressive type of cancer. Differential expression analysis revealed that almost all up-expressed miRNAs in untreated control cells were oncomiR which got down-regulated upon EGCG treatment. In the case of down-expressed miRNAs in untreated control cells, they were identified as tumor suppressor miRNAs which also got up-regulated upon EGCG treatment. NGS study and qRT-PCR validation of selected miRNAs established the potentiality of EGCG in miRNA modulation as well as cancer suppression. Pathway prediction study of selected differentially expressed miRNAs established miRNA as a major regulator of significant breast cancer related pathways. Few suitable diagnostics and prognostics miRNA markers were identified that were having significantly higher as well as differential expression. Nowadays circulating miRNA profiling is in use for early detection of diseases like cancer (Hamam et al., 2017). miRNA inhibitor for highly expressed oncogenic miRNAs can act as a therapeutic regime. Moreover, identified novel miRNAs and their differential expression upon EGCG treatment support the fact that targeting these miRNAs can be a unique therapeutic idea. Small interference RNAs or siRNAs as a drug against diseases have been approved by FDA in 2018 (Kristen et al., 2019), but miRNAs are yet to get the status. On the contrary, natural products against various cancers are taking over the research field. Numbers of studies with EGCG have been shown to enhance the anti-cancerous properties of conventional chemotherapeutic drugs. Various nano-particles or nano-carriers are under study for improvement of the poor bioavailability of free EGCG as well small non-coding RNAs and for the enhancement of its drug-delivery system (Ramesh and Mandal, 2019).

## Data Availability

The datasets presented in this study can be found in online repositories. The names of the repository/repositories and accession number(s) can be found in the article/Supplementary Material.
